# Factors associated with endoscopic full-thickness resection of gastric submucosal tumors

**DOI:** 10.1007/s00464-015-4113-1

**Published:** 2015-04-17

**Authors:** Fei Yang, Sheng Wang, Siyu Sun, Xiang Liu, Nan Ge, Guoxin Wang, Jintao Guo, Wen Liu, Linlin Feng, Wenzhuang Ma

**Affiliations:** Endoscopic Center, Shengjing Hospital of China Medical University, No. 36 Sanhao Street, Shenyang, 110004 Liaoning China

**Keywords:** Gastric submucosal tumor, Endoscopic full-thickness resection, Risk factors

## Abstract

**Objective:**

To identify factors that impact the procedure and treatment outcomes for endoscopic full-thickness resection (EFTR) of gastric submucosal tumors (SMTs).

**Methods:**

Medical records were collected for all patients with gastric SMTs who underwent EFTR procedures in Shengjing Hospital between June 2012 and April 2014. The data from each patient were reviewed, including gender, age, maximum tumor size on endoscopic ultrasound (EUS), tumor location in stomach, length of EFTR procedure, pneumoperitoneum during EFTR, cost to close defects, length of hospital stay after the procedure, and procedure-related complications.

**Results:**

Endoscopic full-thickness resection of gastric SMTs was successfully performed in all 41 patients. Maximum size on EUS [parameter estimate (PE) = 4.443, 95 % confidence interval (CI) 2.191–6.695; *p* = 0.000] and tumor location in the greater curvature (PE = 44.441, 95 % CI 5.539–83.343; *p* = 0.026) were significantly associated with the length of the procedure. A pneumoperitoneum was more likely to occur during EFTR in tumors with a larger EUS size [odds ratio (OR) = 1.415, 95 % CI 1.034–1.936; *p* = 0.03], and less likely to occur during EFTR for tumors located in the posterior wall (OR = 0.003, 95 % CI 0–0.351; *p* = 0.017). The use of the over-the-scope clip (OTSC) system was significantly associated with shorter hospital stays (PE = −1.006, 95 % CI −1.998 to −0.014; *p* = 0.047) and a higher cost of closing defects (PE = 854.742, 95 % CI 358.377–1351.107; *p* = 0.001).

**Conclusions:**

Endoscopic full-thickness resection is an effective and safe method for removing gastric SMTs. Tumor size on EUS and location of the tumor were associated with the duration of EFTR and the occurrence of a pneumoperitoneum during the procedure. The use of an OTSC system was significantly associated with shorter hospital stays and a higher cost of closing defects.

Gastric submucosal tumors (SMTs) are defined as tumors located beneath the gastric mucosa, and include gastrointestinal stromal tumors (GISTs), leiomyomas, schwannomas, malignant lymphomas, lipomas, carcinoids, lymphangiomas, and hemangiomas [1–5]. Gastric SMTs are usually detected incidentally during upper gastrointestinal endoscopy, and have an estimated prevalence of 0.4 % [6–8]. Endoscopic submucosal dissection (ESD) is a minimally invasive endoscopic technique and an important method for gastric SMT resection [9]. With the technical advances in ESD and improvements in endoscopes and accessories over the last decade, endoscopic full-thickness resection (EFTR) was developed to remove large submucosal gastrointestinal lesions [10–14]. Zhou et al. [10] reported the successful use of EFTR in 26 gastric SMTs without laparoscopic assistance, and found that EFTR is an effective, safe, and minimally invasive treatment for patients with gastric SMTs. Several subsequent reports have also described initial experiences with EFTR for SMTs [6, 12, 15, 16]. More recently, the over-the-scope clip (OTSC) system (Ovesco Endoscopy AG, Tübingen, Germany) has been developed as a new device for closing post-operative defects of the gastrointestinal tract. Experimental studies have demonstrated the ability of the OTSC system to close artificial perforations during EFTR [17, 18]. Use of the OTSC system can simplify defect closures during EFTR and result in a more durable closure [19].

Nevertheless, reports on EFTR have been limited to case reports and pilot series. Little is known about the clinical characteristics and risk factors associated with EFTR for gastric SMTs. Therefore, this retrospective cohort study was undertaken to evaluate the impact of various clinical factors in patients with SMTs on EFTR outcomes. To the best of our knowledge, this is the first study to evaluate the clinical factors associated with technical outcomes for EFTR of SMTs.

## Patients and methods

### Study design

This was a retrospective study, the purpose of which was to identify factors that impact the procedure and technical outcomes of EFTR in patients with gastric SMTs. Written informed consent was obtained by all patients before the procedure. This study was approved by the Institutional Review Board of China Medical University in accordance with the Helsinki Declaration.

### Measurements

The medical records of all patients who underwent EFTR for a gastric SMT were reviewed for pertinent clinical information, including age, gender, location of the tumor, maximum size of the tumor, duration of the procedure, development of a pneumoperitoneum during EFTR, method and cost of closing defects, pathologic diagnosis of the tumor, and length of hospital stay after the procedure.

Before EFTR, endoscopic ultrasound (EUS) was performed on all patients to determine tumor size (the maximum diameter), the ultrasonic layer of tumor origin, the echo-texture of the tumor, and the characteristics of neighboring blood vessels. The locations of the lesion were confirmed by abdominal-computed tomography (CT) and gastrointestinal endoscopy. Based on a previous report [20], the locations of tumors in the current study were recorded as gastric fundus and four equal parts in the cross-sectional circumference, including the lesser and greater curvatures and the anterior and posterior walls. All EFTR videos were reviewed to record the duration of the procedure. As the resections were full-thickness, all the patients would experience some degree of pneumoperitoneum. However, pneumoperitoneum caused by small amounts of CO_2_ would be absorbed and relieved quickly. Therefore, in this study, pneumoperitoneum was defined as clinically significant pneumoperitoneum, which was featured as abdominal distention, abdominal percussive tympany, and gastric cavity decompression, and was confirmed by the positive aspiration of gas by abdominal puncture. The cost for defect closure was defined as the total expense of the clips and the OTSC system used in the procedure. All of the removed tumors were paraffin embedded and sectioned for histopathologic and immunohistochemical examinations. Successful resection was defined as *en bloc* resection with negative resection margins and a complete capsule (R0 resection). Procedure-related complications were defined as any newly developed complication after the procedure, such as peritonitis, digestive tract hemorrhage, or local infection. The length of hospital stay after the procedure was defined as the number of days from the day of the procedure to the day that the patient was discharged from the hospital.

### Procedure of EFTR

All EFTR procedures were performed in the operating room with propofol sedation and continuous cardiorespiratory monitoring. EFTR was performed as previously described [10]. All of the EFTR endoscopy procedures were performed by one endoscopist. A CO_2_ insufflator was used during the procedure. EFTR was performed without laparoscopic assistance; however, if persistent bleeding or injury to an adjacent organ occurred, the procedure was converted to a laparoscopy. The tumor, including the surrounding mucosa, muscularis propria, and serosa, was completely removed without injury to the tumor capsule in all cases. The post-resection gastric defect was closed immediately using metallic clips or an OTSC system. A 20-gage needle was inserted into the peritoneum via the right lower quadrant during the procedure in the patients with clinically significant pneumoperitoneum.

### Endoscopic equipment and accessories

All of the procedures were performed with high-definition endoscopes and EPK-i processors (Hoya, Tokyo, Japan). A transparent cap was attached to the front of the endoscope. Hook and IT knives were used to dissect the submucosal layer and peel the tumor. A high-frequency generator was used with the Hybrid Knife system (Erbe Elektromedizin, Tübingen, Germany). Other equipment included injection needles, grasping forceps, snares, hot biopsy forceps, metal clips, and an OTSC system. In the current study, two types of metal clips were used, including a small clip (HX-610-135L; Olympus, Tokyo, Japan) with a maximum jaw span of 9 mm and a big clip (Resolution™; Boston Scientific, Boston, MA, USA) with a maximum jaw span of 11 mm. The small clips (HX-610-135L) were used to close smaller defects. Big clips (Resolution) were used to close defects that were bigger and more difficult to close because its jaw span was bigger and it could be switched on and off repeatedly. The use of OTSC was similar to that of big clips, and only in the patients who could afford the cost of an OGTC system.

### Statistical analysis

Categorical variables are expressed as frequencies and percentages. Continuous variables are expressed as the mean and standard deviation (SD). The independent variables used in the models included gender, age, size on EUS, tumor location, and OTSC. The technical outcomes included duration of the procedure, a pneumoperitoneum during EFTR, length of hospital stay, cost of defect closure, and complications. Multivariate linear regression was used to assess the relationship between the clinical factors and treatment outcomes (when outcomes were numerical, continuous data). Logistic regression (when outcomes were categorical data) was used to test for effect associations among outcomes and independent variables. All reported *p* values were two tailed, and *p* values <0.05 were considered to indicate statistical significance. Statistical analysis was performed using SPSS 17.0 software (SPSS, Chicago, IL, USA).

## Results

The current study included 41 patients who underwent EFTR for gastric SMTs between June 2012 and April 2014 at Shengjing Hospital of China Medical University. The patient characteristics and treatment outcomes are summarized in Table 1. There were 13 (31.7 %) males and 28 (68.3 %) females enrolled in the study. The mean age of the patients was 53.95 ± 14.10 years. All of the patients underwent EUS; the mean maximum tumor size based on EUS was 16.34 ± 5.89 mm. Of the 41 SMTs, 1 (2.44 %) was located in the anterior wall of the antrum, 2 (4.88 %) in the greater curvature of the antrum, 6 (14.63 %) in the anterior wall of the corpus, 6 (14.63 %) in the greater curvature of the corpus, 4 (9.76 %) in the lesser curvature of the corpus, 9 (21.95 %) in the posterior wall of the corpus, and 13 (31.71 %) in the fundus of the stomach.

**Table 1:** The patient characteristics and treatment outcomes

Variable	Mean ± SD (*n*, %)
No. of patients	41
Age (years)	53.95 ± 14.10
Gender (male)	13 (31.7 %)
Size of tumor (mm)	16.34 ± 5.89
Location of tumor	
Antrum anterior wall	1 (2.44 %)
Antrum greater curvature	2 (4.88 %)
Corpus anterior wall	6 (14.63 %)
Corpus greater curvature	6 (14.63 %)
Corpus lesser curvature	4 (9.76 %)
Corpus posterior wall	9 (21.95 %)
Fundus	13 (31.71 %)
Duration of EFTR	78.82 ± 46.44
OTSC	6 (14.63 %)
Pneumoperitonea during EFTR	26 (63.41 %)
Cost of defect closing	1014.04 ± 524.89
In-hospital days	5.39 ± 1.14
Pathology	
Carcinoid tumor	1 (2.44 %)
GIST	33 (80.49 %)
Heterotopic pancreas	1 (2.44 %)
Hyaline degeneration	1 (2.44 %)
Leiomyoma	4 (9.76)
Schwannoma	1 (2.44 %)
Complications	9 (21.95 %)
Abdominal pain	2 (4.88 %)
Dysuresia	1 (2.44 %)
Abdominal pain and fever	3 (7.32 %)
Nausea and vomiting	1 (2.44 %)
Pharyngalgia	1 (2.44 %)
Tenderness of upper abdomen	1 (2.44 %)

*OTSC* over-the-scope clip system, *GIST* gastrointestinal stromal tumor

Endoscopic full-thickness resection was successfully performed endoscopically without laparoscopic assistance in all patients. All of the artificial perforations were tightly closed. All of the pneumoperitoneums were decompressed by abdominal puncture and endoscopic suction. An OTSC system was used in six (14.63 %) patients; metal clips were used in 35 (85.37 %) patients. The final pathologic analyses revealed that all 41 resection specimens included all gastrointestinal tract wall layers without positive margins or capsule rupture (R0 resection). Pathologic examination determined that 33 tumors (80.49 %) were GISTs, four tumors (9.76 %) were leiomyoma, one (2.44 %) of the tumors was a carcinoid, one mass (2.44 %) was a heterotopic pancreas, one mass (2.44 %) was hyaline degeneration, and one tumor (2.44 %) was a schwannoma.

Table 2 summarizes the characteristics associated with the duration of the procedure, pneumoperitoneum during EFTR, length of hospital stay, cost of defect closure, and complications. The mean duration of the procedure was 78.82 ± 46.44 min. Based on multiple logistic regression analysis, EUS size and tumor location were significantly associated with the duration of the procedure. For every 1 mm increase in EUS size of the tumor, the estimated duration of the procedure increased by 4 min [parameter estimate (PE) = 4.443, 95 % confidence interval (CI) 2.191–6.695; *p* = 0.00]. Tumors located in the greater curvature required more time for resection (PE = 44.441, 95 % CI 5.539–83.343; *p* = 0.026) compared with tumors in the other regions of the stomach. Pneumoperitoneums occurred in 26 patients (63.41 %) during EFTR. Pneumoperitoneums were more likely in patients with larger tumor sizes [odds ratio (OR) = 1.415, 95 % CI 1.034–1.936; *p* = 0.03], and less likely in patients with tumors located in the posterior wall (OR = 0.003, 95 % CI 0–0.351; *p* = 0.017). The mean length of hospital stay for all patients was 5.39 ± 1.14 days. The use of an OTSC system was significantly associated with a shorter hospital length of stay (PE = −1.006, 95 % CI −1.998 to −0.014; *p* = 0.047). The mean cost for closing defects during EFTR was 1014 ± 524.89 USD. The OTSC system was associated with a higher cost to close defects (PE = 854.742, 95 % CI 358.377–1351.107, *p* = 0.001).

**Table 2:** Multivariate regression analysis for procedure duration, pneumoperitoneum during EFTR, length of hospital stay, cost of defect closure, and complications

Characteristics	Procedure duration, PE 95 % CI	Pneumoperitoneum, OR 95 % CI	Hospital stay, PE 95 % CI	Cost of defect closure, PE 95 % CI	Complications, OR 95 % CI
Gender	−1.228 (−28.947 to 26.49)	0.089 (0.003 to 3.175)	0.109 (−0.648 to 0.866)	−245.643 (−624.433 to 133.146)	4.901 (0.623 to 38.558)
Age	−0.18 (−1.004 to 0.643)	0.996 (0.892 to 1.111)	−0.006 (−0.028 to 0.017)	0.13 (−11.121 to 11.38)	1.025 (0.962 to 1.092)
Size in EUS	4.443 (2.191 to 6.695)*	1.415 (1.034 to 1.936)*	−0.001 (−0.062 to 0.061)	−0.448 (−31.222 to 30.325)	1.226 (0.978 to 1.537)
Anterior wall	25.479 (−11.329 to 62.288)	–	0.81 (−0.195 to 1.815)	353.475 (−149.526 to 856.477)	–
Posterior wall	−0.526 (−36.532 to 35.48)	0.003 (0 to 0.351)*	−0.882 (−1.865 to 0.101)	−45.931 (−537.97 to 446.108)	–
Greater curvature	44.441 (5.539 to 83.343)*	–	0.699 (−0.363 to 1.762)	370.663 (−160.946 to 902.272)	–
Lesser curvature	20.21 (−21.938 to 62.359)	–	0.599 (−0.552 to 1.75)	100.092 (−475.881 to 676.066)	–
OTSC	9.405 (−26.917 to 45.728)	0.677 (0.024 to 18.814)	−1.006 (−1.998 to −0.014)*	854.742 (358.377 to 1351.107)*	0.228 (0.011 to 4.562)

*OR* odds ratio, *PE* parameter estimate, *CI* confidence interval

* p < 0.05

There were no cases of bleeding, peritonitis, or abdominal abscesses. Based on multiple logistic regression analysis, there were no significant factors associated with procedural complications. Procedural complications included abdominal pain [*n* = 2 (4.88 %)], dysuresia [*n* = 1 (2.44 %)], nausea and vomiting [*n* = 1 (2.44 %)], pharyngalgia [*n* = 1 (2.44 %)], tenderness of the upper abdomen [*n* = 1 (2.44 %)], and fever [*n* = 3 (7.32 %)]. Abdominal pain, nausea and vomiting, pharyngalgia, and tenderness of the upper abdomen were self-limiting, and resolved within 3 days. Fever was managed with antibiotics and proton pump inhibitors, and resolved within 1–3 days. Dysuresia was managed with urine tube placement.

## Discussion

The EFTR technique for gastric lesions was first described by Suzuki and Ikeda in 2001 [13]. With respect to the uncertainty of endoscopic closure for a large perforation and the potential risk of intraperitoneal infection, EFTR has most often been used in conjunction with laparoscopy in the last decade. In 2008, Hiki and colleagues [21] described this combination for the local resection of GISTs. Tsujimoto et al. [22] reported satisfactory surgical outcomes of endoscopy combined with laparoscopy for gastric SMTs. Abe et al. [23] reported that laparoscopy-assisted EFTR is an effective approach for select patients with gastric SMTs; however, the disadvantages of EFTR combined with laparoscopy, including a large surgical wound, high medical cost, and long hospital stay, cannot be ignored. Most gastric SMTs, including GISTs, usually grow without restraint in the gastric wall and rarely metastasize to the regional lymph nodes [24, 25]. Surgical procedures for GISTs do not require lymphadenectomy or wide surgical margins. Thus, surgeons should strive to make the smallest resection as practical in treating gastric SMTs [23]. Zhou et al. [10] first reported the successful use of EFTR in 26 patients with gastric SMTs without laparoscopic assistance in 2011, and reported satisfactory results. Shi [12] provided a novel method for repairing gastric defects resulting from EFTR using metallic clips and endoloops. Schlag et al. [6] found EFTR to be possible for tumors with diameters <3 cm and clear intraluminal growth.

In the present study, all of the tumors were successfully resected using EFTR. No severe complication occurred. All of the complications related to the procedure were self-limiting or resolved within 3 days. We were unable to identify any significant factor associated with the complications.

The sizes and locations of the tumors were associated with the duration of the procedure and the occurrence of a pneumoperitoneum during EFTR. It is likely that a larger tumor is more difficult to resect using endoscopy. The larger defect in the gastrointestinal wall after tumor removal and a longer operative time might result in an increased risk for a pneumoperitoneum. It is interesting that our results showed that the tumor location was also associated with the duration of the procedure and the occurrence of a pneumoperitoneum. For tumors located in the greater curvature, the procedure duration was longer than tumors located in other regions of the stomach. For tumors located in the posterior wall, the risk of a pneumoperitoneum was lower. We considered that this difference was mainly caused by different anatomic structures external to the stomach. The posterior wall of the stomach is the anterior wall of the lesser sac. Perforation of the posterior wall into the lesser sac will be confined and less gas and/or gastric fluid will escape into the peritoneal cavity. The structure adjacent to the greater curvature is the peritoneal cavity. After an intentional perforation is made in this part of the stomach, air and fluid escape into the peritoneal cavity. The pneumoperitoneum is formed. The endoscopic view will be limited because of the decompression of the stomach. Defect closure will be challenging in cases with an unsatisfactory endoscopic view, which may lead to a longer operative time for tumors located in the greater curvature. Furthermore, as there is no support structure posterior to the greater curvature, the mobility of this part of the stomach is larger than the other parts. As a result, the difficulties of the procedure might be larger in the greater curvature than in the other parts. Moreover, the potential risk of contamination to the peritoneal cavity and peritoneal infection cannot be ignored in patients with the tumor located in this part of stomach [26].

According to previous reports [27–29], tumors located in the anterior gastric wall and greater curvature of the stomach can be easily resected via laparoscopy with minimal invasion. In contrast, lesions on the posterior gastric wall and lesser curvature are not always easy to reach. Surgeons need to open the gastrocolic ligament with ultracision to inspect the posterior wall of the stomach. In the present study, however, we found the opposite situation; it was faster and safer to remove tumors located in the posterior gastric wall than the greater curvature, which suggests that SMTs located in the posterior gastric wall may be more suitable for EFTR.

In the current study, an OTSC system was used in six patients. The use of an OTSC system was associated with a shorter hospital stay. There was no significant difference in the duration of EFTRs in which an OTSC was or was not used. The OTSC system was also significantly associated with a higher cost to close the defect.

Our study had limitations. First, the study was a retrospective study. Second, the study was a single-center study with a small number of patients. Third, we have not evaluated the long-term clinical outcomes. After resection, the follow-up may have occurred in another hospital, and review of the results was challenging. All of the above factors are important points to evaluate in a prospective study with a larger sample size.

## Conclusions

EFTR is effective and safe for gastric SMTs. EUS size and location of the tumor were associated with the duration of the procedure and the occurrence of pneumoperitoneums during EFTR. The use of an OTSC system was significantly associated with a shorter hospital stay and a higher cost of defect closure.
